# Nitrous Oxide and Oxygen Anesthesia

**Published:** 1911-10-15

**Authors:** E. P. Dameron

**Affiliations:** St. Louis, Mo.


### NITROUS OXIDE AND OXYGEN ANESTHESIA.

BY E. P. DAMERON, D.D.S., ST. LOUIS, MO.

Read before the Tennessee State Dental Association, May 25, 1911.

Glittering and attractive is the fame and adulation given to the world's heroes. The orator, who with matchless eloquence sways our minds and our hearts, possesses a transcendent gift. The sculptor who carves forms to elevate and instruct is held to be gifted of God. The painter whose canvas breathes smiles and sunshine or depicts tragedy and care is in high reverence. The musician who weaves a spell as composer or interpreter gathers our heartstrings in his grasp bringing tears or laughter as we bow to his wand. But there comes to us to-day an insistent thought which clamors for audience and which says to us that the reward of the scientist who has by his laborious investigations arrived at valuable and important facts and made them into workable form has gained a place in history which will be acknowledged long after heroes, sculptors, painters and musicians are forgotten.

The scientist who unearthes riches and by that adds to the sum total of adaptable truths by which human life expands and human misery is alleviated makes for himself a monument builded by his fellow-men—he hath no enemy—the rich, the poor, the high and the low alike do him honor. From 1700 to 1800 there was a man who lived to a ripe old age, and during his life he became well known as a politician and as a diplomat: he also did the wonderful thing—he identified lightning and electricity. Would you not rather be known as Benjamin Franklin the scientist than Benjamin Franklin the diplomat?

Early in the 18th century, about the time of the War of 1812, a plodding scientist in Penzance, where the English collieries were largely located, was struck with the enormous fatality to those who went into the bowels of the earth to wrest from the subterranean passages the means of light and heat. Mysterious explosions, alarmingly frequent, and the miners at the mercy of the unknown foe. Our plodding scientist assiduously bent himself to the task of unraveling the mystery with the result that "Fire Damp" was explained and the "Safety Lamp" was given to the world and the name of Sir Humphrey Davy shines resplendent throughout the land. Now the wives and children or the sweet hearts of the miners bid them good-bye at the mouth of the pit without the former dread that they may wring their hands at nightfall. Would you not, be the scientist Humphrey Davy rather than Michael Angelo? I am sure you would, and so would I.

Following Priestlys discovery of the method of liberating and collecting gases there was established at Bristol, England in 1798 an institution for research-work along that line and there was placed in charge as Superintendent Sir Humphrey Davy who suggested that nitrous oxide gas might be used for relief of pain but not until 1844 was this prophesy demonstrated a reality by Wells, and suffering humanity's greatest boon—anesthesia— was given to the world and the world acknowledged its debt when on March 27th, 1910, at Paris, France, a monument to Horace Wells was unveiled. Oliver Wendell Holmes, who gave the name of "anesthesia" to the state produced, once said, "Science is a first rate piece of furniture for a man's upper chamber, if he has common sense on the ground floor—but if he hasn't plenty of good common sense, the more science he has the worse for the patient." (This is especially true in producing anesthesia with nitrous-oxide—the operation requiring about as much common sense as it does gas.) With in the lifetime of some of you—1868—the combining of oxygen with nitrous oxide in producing anesthesia was introduced. Experimented with and studied thoroughly by Dr. Paul Burt of France ten years afterward, and later, brought to practical use by Dr. F. W. Hewitt of London.

Burt and Hewitt agreeing that N2O was a true anesthetic and that the patient was anesthetized not asphyxiated as was at first thought to be the case when N2O was administered. "Anesthesia can be maintained for hours by the addition of air or oxygen, this proving conclusively that nitrous oxide possesses anesthetic properties of its own." Results have shown that air lessens somewhat the asphyxiation symptoms of nitrous oxide but if sufficient air is admitted to overcome all asphyxiation the nitrous oxide loses its anesthetic effect, whereas varying proportions of oxygen properly given, eliminate cyanosis without lessening anesthesia.

According to Teeter nitrous oxide on entering the lungs is distributed throughout their alveoli and is taken up by the blood and passes to the lymph. As the nitrous oxide comes in contact with the delicate nerve cells, an action occurs that causes a temporary cessation of their functional integrity. The most highly organized centers are first affected the others finally all succumb and respiratory and cardiac centers become paralyzed, if the anesthesia is pushed to fatality. There does not seem to be any great danger in the administration of nitrous oxide and oxygen but the condition produced is not a natural one and the anesthetist should at all times exercise the greatest of care and be constantly on the alert for unfavorable symptoms. Some one has said that "Men who are dealing with human life must have minds clear as crystal, strong as steel, yet shades of thought as soft as shadows; at times, a firm resolute touch and again as delicate as the velvet touch of angel fingers." As we give careful attention to the physical and mental condition of our patients so we should attend to our own physical and mental condition.

Beginning with Wells, studied and improved by Paul Burt, Frederick W. Hewitt, Chas. K. Teeter, W. H. De Ford and many others we have within the confines of our own profession developed an anesthetic that is practically free from danger that any one competent to practice dentistry should be competent to administer whenever its use is indicated. The fear of pain prevents a large number of those needing dental treatment from attending to such treatment and untold havoc is wrought—teeth lost, digestion ruined, nerves shattered and often life itself is menaced; and that fear is not altogether unfounded. Year in and year out, so long "That the mind of man runneth not to the contrary," we have gone on inflicting peculiar and exquisite torture that has become synonomous with the name dentist and that would not in this day and age be tolerated in any other branch of the healing art. But, the world moves, we are improving—are becoming more humane—"painless dentists" are springing up all over the land and while that term now provokes a smile, it is not beyond the realm of reason.

With this somewhat lengthy prologue, I desire to briefly mention a few important points in the administration of nitrous oxide and oxygen. It is not necessary to describe apparatus other than to say it consists of cylinders, holder and bags for both nitrous oxide and oxygen, a mixing and a warming chamber and connecting tubing, but I do desire to direct especial attention to the nasal inhaler designed by Dr. Teeter. Since acquiring it, I have done away with the face inhaler except in those cases where nasal breathing was impossible. After adjusting the inhaler and inserting the mouth prop and filling both bags about two-thirds full, I instruct the patient to exhale—blow out—knowing that the following inhalation will be a good deep one, after two or three such "breaths" with air only, I turn off the air and admit nitrous oxide and place a strip of rubber over the mouth, continuing the admission of nitrous oxide only, until some slight ,cyanosis appears, then gradually admitting oxygen to restore normal color. If for extracting, I proceed to deep anesthesia, if for operative procedure I carry just to the anesthetic state for the initial work and as the operation proceeds, bring them back into semiconscious or analgesia state having repeatedly assured the patient there would be no pain. Having secured the desired state of anesthesia or analgesia, I release the spring in the upper part of the valve of the inhaler thus causing the patient to breathe to and fro—into the bags. There may be some of you who object to the rebreathing of the contents of the bags and in defense of that method I quote from an article in the Journal of the American Medical Association, by Dr. W. D. Gatch, referred to in an editorial in the *Dental Summary.*

"The question now arises whether rebreathing in the manner just described is injurious. Many writers have attached great importance to certain volatile organic poisons in the expired air. This has caused several inventors of apparatus for giving nitrous oxide and oxygen to do away entirely with rebreathing. Hewitt's apparatus, although its inventor states that rebreathing is probably not harmful—does not permit rebreathing. Dr. H. Warren Buckler, anesthetist for Dr. H. A. Kelly has used the method of rebreathing for several years and has noted no ill effects therefrom. Rebreathing provided enough oxygen be given to prevent cyanosis can be permitted for a surprisingly long time without any appreciably injurious results. According to Henderson, carbon dioxide has a direct stimulant action on the vasometer center. The carbon dioxide is not the only product of expiration, the effect of which must be studied with regard to rebreathing. The heat of the exhaled gases has also an important action. Normally the body loses a large amount of heat in the expired air. This loss is prevented by rebreathing. Therefore there is a rise of body temperature. Rebreathing is thus a most effective method of heating the gases which are cold on being freed from their cylinders. Besides being safe and convenient, this form of anesthesia has, I believe, a unique characteristic which makes it especially valuable for very sick patients. I refer to its remedial action for shock. This effect may make of the anesthetic an aid instead of an evil. With absolute control of the patient it is surprising how little time it takes to open up a tooth, remove a pulp or properly prepare cavity for filling.

DeFord says in reply to the query,—In what class of cases could nitrous oxide and oxygen be used? "In all painful conditions the dentist is called upon to treat: Sensitive cavity preparation, removal of pulps, shaping teeth for crowns or abutments whether alive or devitalized, adjusting cervical or painful clamps, treating pyorrhea, rapid wedging of the teeth to gain space for filling, opening into teeth affected with pericementitis or acute alveolar abscess, lancing abscesses—in short all painful or fatiguing operations on the teeth. Once familiar with operating under anesthesia, you would relinquish dentistry rather than practice as you are now doing."

I readily admit many of the foregoing conditions can be operated upon with very little pain, sharp burs, chisels, anodynes, a careful hand, etc., are well enough and will usually answer the purpose but at best there is always some pain and a nervous patient can easily and usually does magnify that little pain into one of great magnitude. It is for these cases and persons that I urge upon you the efficacy of this anesthetic. "But," you will say, "the anesthetic is expensive;" that is true, but I have found little difficulty in that regard and my apparatus is a source of income rather than expense. John Ruskin said, "A man or woman in public or private life, who ever works only for the sake of the reward that comes for the work will in the long run do poor work always. I do not care where the work is, the man or woman who does work worth doing is the man or woman who lives, breathes and sleeps that work; with whom it is ever present in his or her soul; whose ambition is to do it well and feel rewarded by the thought of having done it well. That man, that woman, puts the whole country under an obligation."

References: *Dental Cosmos, Dental Summary, DeFord's Anesthesia in Dentistry, J. D. Patterson, Chas. K. Teeter.*

### DISCUSSION.

Dr. R. Boyd Bogle, of Nashville: I wish to express my appreciation of the Doctor's most excellent paper. There is one thing about nitrous oxide anesthesia that I have noticed, and that is that we do not get as much relaxation as we might desire. I notice that in surgical operations there seems to be a rigidity of the muscles, and especially in dental operations, we have difficulty in getting the mouth open, and we lose a good deal of time unless we continue the administration of the nitrous oxide, as described by Dr. Dameron. Before the nose piece was introduced the anesthetist had to put the patient to sleep, remove his apparatus and go to work, but the nasal inhaler has very largely overcome that difficulty. Before the introduction of the use of oxygen in addition to the use of nitrous oxide and the use of air, as the doctor has stated, there was noticed a cyanotic condition, due to the lack of oxygen, the patient became black in the face to some extent, and there was so much discoloration of the blood that it was difficult for the operator to tell what conditions were present, and to determine what tissues were diseased owing to the discoloration. These conditions, in some instances, were removed almost entirely by the addition of oxygen.

Now as to the length of the operation, I believe that the dentist should be a surgeon, and where you have a long tedious operation such as the preparation of cavities, cutting of pulps that are not exposed, extracting a great many teeth that are broken down, or removing the pulp, or some such operation, the anesthetist should look after that if we are going to do the operation, because a man has enough to do in performing the operation properly, either operation is sufficient to keep one man busy. As the Doctor has said a man must have a great deal of common sense, he must be clear headed, he must have the touch of an angel, he must have a strong will, otherwise he will be up in the air. A certain doctor came to me and was discussing the use of nitrous oxide for the purposes of surgical work; I told him that he should be careful with nitrous oxide, because you must remember that you have a patient who may be injured He said that he had given it possibly fifty or sixty times, and I told him to be careful with it, and not to forget that it is dangerous just like chloroform or ether. He said, "Don't you worry about me;" and I said, "I am not worrying about you, I had rather worry about the patient." It wasn't four days after that until he came to me and I said, "What are you doing?" and he said, "I nearly killed a patient yesterday with that stuff." I said, "What were you doing, watching the operation?" and he said, "I guess I was." He had gotten his mind off the business for a moment at the expense of the patient.

Now the Doctor mentions one thing that the surgeons and dentists used to talk about in the chloroform operation, that more patients died of collapse in the dental chair than almost any other place, because of the position and the shock to their nerves. As we proceed to work with nitrous oxide cases, you can have the patient near enough to consciousness to carry on a conversation, so there certainly must be some action of the gas that takes care of that; but if I was going to cut into a live pulp, I would want that patient thoroughly anesthetized, I would want him to sleep good.

There is one other thing I wish to speak of and that is the effect of the gas upon some patients. The safety of nitrous oxide is remarkable. Some dentists administer the gas until the patient gets black in the face, then they throw the inhaler down and go to extracting teeth. I say some dentists, they claim to be dentists, painless dentists, and they have their signs on the colored sides of buildings, but I have been inquiring around and they sometimes have bad after effects, in fact a doctor told me not long ago that he had been called to a certain place in this city, and that he had had considerable trouble with the patient. I recall a case of a girl here in this city, who was put under the influence of the gas, and she was in a very bad condition for three or four hours. Neither the dentist nor his assistant took the patient's name or address, and they didn't know who she was or where she lived; and it seemed that her people didn't know where she had gone to have her teeth extracted; it was probably about 8:30 at night before they could find out where she lived or send her home in a carriage. And I want to say too that that dentist had been using nitrous oxide for a long time, and he had the mixture of oxygen in addition to the nitrous oxide gas. For children I believe we have an ideal anesthetic. I am not prepared to say whether the addition of oxygen will keep down the jactation of the body and limbs. I have not found the relaxation of the nitrous oxide to be as much as under the other anesthetics.

Dr. Sevier: I want to thank the essayist for that paper. It was very interesting to me. I have a reputation down in my town of being a killer. He says he prepares teeth for crowns with it. I want to ask him if he keeps the patient under the influence of it for twenty or thirty minutes, it will take that long to do the work.

Dr. J. W. Bryan: I think the essayist has given us a splendid paper and it is not necessary to extend thanks every time I think, but we all appreciate it. I think one of the most important points that he spoke of was that the operator or anesthetist, should keep his head clear, and watch his business, or attend to his duties. I think this is the source of the greatest evil, he forgets what he is doing, his mind is on other things and not on the nitrous oxide. We find there are peculiar individual idiosyncracies that we know absolutely nothing about until we run on them. For instance I had a patient a few years ago to whom nitrous oxide had been administered some two or three years previously. She came to me to have a tooth removed. She said, "Do you use gas?" And I said, "That depends on the case." She replied, "I

don't take any." I asked, "Why?" She remarked, "I took some three or four years ago and ever since that I have been wandering as a child lost in the wilderness, I have never yet been able to relocate myself." I would like to ask the essayist if he has had any experience like that. This lady was about twenty-four or five years old, she must have been in the neighborhood of twenty or twenty-one when she took the gas. The operation was successful. Nothing at that time was observed. I was not the man who administered the gas, and was not present when it was administered, and I did not know the lady when this happened, but she says that a kind of lost feeling is over her all the time; if you can imagine yourself lost and wandering about through life that is the sensation she feels. But as I said, I think a great deal depends upon the common sense and clear thinking of the one who is anesthetizing, and not having his work divided up so as to have to look after more than one thing at a time.

Dr. Bates: It has been so long since I have given nitrous oxide I feel I am getting rusty, I do not generally have any demand for it in Shelbyville. Occasionally I do have occasion to use it, but not often. I have given nitrous oxide a number of times but never heard of a case as just described by Dr. Bryan, except the dreams are very vivid under nitrous oxide, and some times for a short time afterwards it is almost impossible to disabuse the mind of the patient that the dream is not an actual occurrence. It has been my experience with nitrous oxide that there is a kind of hallucination, that the dream is even more vivid in the mind of the patient and more difficult, I think, to relieve the patient of, than in either ether or chloroform. Perhaps in this patient that the Doctor has just referred to, it is an auto-hypnotic condition that the patient had produced by a vivid dream. I have only had one case of cessation of breathing in nitrous oxide, and that was a very aenemic young woman who had just returned from a long period in the hospital, and she suddenly stopped breathing without showing any cyanosis. I had a very good physician assisting me at the time, and he gave her a few whiffs of oxygen, and she began to breathe in about two minutes. That is the only case that I ever heard of or saw that the patient actually stopped breathing under the administration of nitrous oxide. I would like to know of some other cases somebody else has seen. There are some recorded, but I have never seen another.

Dr. Rudolfh: Last September I happened to be at the hospital and Dr. Haggard asked me to assist him in giving nitrous oxide to a patient for an abdominal operation, and she was kept under the gas for an hour and twelve minutes. He failed to get a proper relaxation at one stage of the anesthesia and we added ether. Now whether that would be applicable in oral surgery, I do not know, but it acted very nicely in that operation.

Dr. Dameron: I thank you very much for the discussion of the paper. I have given it in cases of all kinds, heart trouble, kidney trouble, etc. I have had a few little scares, but nothing serious, I have only administered it some 520 times I believe, but as to the length of time, I have never kept the patient under it longer than forty-five minutes, but I have been with Dr. Teeter of Chicago, and I have seen him keep them under it for from one to four hours in surgical operations.

The question of Dr. Bryan's as to the patient some three or four years afterwards, I would suggest that is not due to the gas especially, it must have been a hypnotic condition as DeFord tells us in his work, you make suggestions to the patient that they are going to sleep nicely, and he says that lots of times they are hypnotized as well as anesthetized. Something of that kind may have occurred in this case. I could relate many cases that I have heard of or read of, but they never have scared me. I always have a mouth prop in place, and sometimes after administering the gas the prop will drop out, showing a relaxation. Sometimes the mouth will close, but it would open again by increasing the anesthesia.

